# Political ideology and generosity around the globe

**DOI:** 10.1073/pnas.2219676120

**Published:** 2023-04-05

**Authors:** Veronica Pizziol, Xhiselda Demaj, Roberto Di Paolo, Valerio Capraro

**Affiliations:** ^a^Institutions, Markets, Technologies School for Advanced Studies Lucca, Lucca 55100, Italy; ^b^Analysis of Complex Economic Systems Unit, Department of Economics, Ca’ Foscari University of Venice, Venice 30121, Italy; ^c^Department of Psychology, University of Milan Bicocca, Milan 20126, Italy

**Keywords:** political ideology, generosity, COVID-19, quality of governance

## Abstract

In a world severely put under stress by COVID-19, generosity becomes increasingly essential both when able to transcend local boundaries, building upon universalistic values, and when directed toward more local contexts, such as the native country. This study aims to investigate an underresearched determinant of generosity at these two levels, a factor that captures one’s beliefs, values, and opinions about society: political ideology. We study the donation decisions of more than 46,000 participants from 68 countries in a task with the possibility of donating to a national charity and an international one. We test whether more left-leaning individuals display higher generosity in general (H1) and toward international charities (H2). We also examine the association between political ideology and national generosity without hypothesizing any direction. We find that more left-leaning individuals are more likely to donate in general and more likely to be generous internationally. We also observe that more right-leaning individuals are more likely to donate nationally. These results are robust to the inclusion of several controls. In addition, we address a relevant source of cross-country variation, the quality of governance, which is found to have significant informative power in explaining the relationship between political ideology and the different types of generosity. Potential mechanisms underlying the resulting behaviors are discussed.

Political ideology is a set of beliefs, values, and opinions about society’s proper order (1). In its most contemporary meaning, political ideology can be defined over three dichotomies, i.e., left–right, libertarianism–authoritarianism, and pluralism–populism (2). This paper focuses on the left–right distinction, which has been the most studied and is appropriate for cross-cultural comparisons (3). For example, a meta-analysis of 88 studies in 12 countries found that the left–right ideological self-placement covaries with social psychological variables concerning uncertainty and threat, such as death anxiety, fear of loss, personal needs for order and closure, or personality traits, such as openness and conscientiousness (4). Typically, left-wing ideologies promote social change and are egalitarian and liberal, while right-wing ideologies favor the conservation of the *status quo* and hierarchy. The left–right distinction does not only map onto different visions of the favored social order but also has many practical implications, being associated with environmentalism (5), the ability to discern fake news (6), and, more recently, COVID-19 infections (7).

This study examines the association between political ideology and two types of generosity: One type has its roots in localism and captures the intention to be generous at the national level; the other, more universalistic, measures the willingness to be helpful at the international level. In an increasingly globalized world, this type of generosity, able to transcend local boundaries, becomes of even greater importance and consequently does understanding its determinants. Liberals, compared to conservatives, have been shown to be more motivated to sacrifice their self-interest for mutually beneficial outcomes (8) and to identify more with the world as a whole (9), as well as to have a universalist mindset (10). Hence, we hypothesize that left-leaning individuals may display higher generosity in general (H1) and toward charities operating internationally (H2) compared to right-leaning individuals. We do not hypothesize any direction for the association with national generosity in light of recent findings (11), showing that liberals are more generous than conservatives to their nation, which contrasts expectations based on stronger nationalism displayed in more conservative individuals.

We employed a global dataset collected in April–May 2020, containing 51,402 unique observations from 69 countries, from all continents but Antarctica (12, 13). The samples were recruited by national teams, with the goal of obtaining, when possible, nationally representative samples with respect to sex and age. (*SI Appendix* for details about recruitment). Our measure of political ideology resembles the one used in ref. 4: Subjects were asked to identify their political orientation on a scale from 0 (very left leaning) to 10 (very right leaning). Our measure of generosity refers to charity organizations working to protect people from COVID-19. It is measured as the proportion of the daily wage in the corresponding country that participants keep for themselves (*Self-interest*), give to a national charity (*National generosity*), or to an international charity (*International generosity*). These measures are available for more than 46,000 participants in 68 countries.

Related to our study, ref. 9 showed, over a sample of 18,411 participants from 42 countries, that liberals cooperate slightly more than conservatives in a prisoner’s dilemma. Compared to it, we innovate in two ways. First, we consider measures about giving, which entail only preferences, rather than cooperation, which also involves beliefs. Second, ours is a COVID-19-related study and thus brings evidence on prosocial behavior in emergencies, when it is needed the most. Most recently, ref. 11 studied the link between political ideology and generosity at different administrative levels in the United States and Italy, finding that liberals, compared to conservatives, are more likely to be generous at the national and global levels, while they are similar at the state/regional level. Compared to it, we can draw more general conclusions given the global coverage of our dataset, and we can study the heterogeneity of the effect through country-specific factors.

In line with our predictions, we find that i) more left-leaning individuals are more likely to donate in general and ii) more likely to be generous internationally. Also, while we were agnostic on donations at the national level, we do find that more right-leaning people are more likely to donate nationally. These findings are robust to the inclusion of several controls, including COVID-19 conspiracy beliefs, thus ruling out the explanation that right-leaning people tend to be less generous toward COVID-19 charities because they are less likely to believe in COVID-19.

Last, we study heterogeneity across countries, focusing on an indicator of the quality of governance which has been found to be associated with individualist vs universalist values (14). Related to our work, Romano et al. (9) found that *government effectiveness* and *rule of law* moderate the association between political ideology and parochial cooperation as well as general cooperation. We find that these moderations generalize to several other measures of quality of governance and to the relationship between political ideology and generosity. This allows us to propose a theoretical mechanism to explain our findings.

## Results

The first three columns of Table 1 report linear regressions predicting self-interest, national generosity, and international generosity as a function of political ideology. More right-leaning individuals are associated with higher self-interest (*β* = 0.083, *t* = 11.58, *P* <  0.001) and lower international generosity (*β* = −0.086, *t* = −18.51, *P* <  0.001), whereas there is no significant correlation between national generosity and political ideology (*β* = 0.001, *t* = 0.06, *P* = 0.95). Taking into account the hierarchical nature of the data by adding country fixed effects, which returns the same outputs as a multilevel mixed-effects model used in previous studies (9, 13), columns (4) to (6) confirm the positive association between right-leaning ideology and self-interest (*β* = 0.042, *t* = 5.95, *P* <  0.001) and the negative correlation between right-leaning ideology and international generosity (*β* = −0.062, *t* = −13.88, *P* <  0.001). It also discloses a positive relationship between right-leaning ideology and national generosity (*β* = 0.018, *t* = 2.99, *P* = 0.003). Columns (7) to (9) include both country fixed effects and a set of controls (gender, age, employed, student, collective narcissism, national identity, individual narcissism, moral circle, moral cooperation, open-mindedness, COVID-19 conspiracy beliefs, health condition, and self-ladder) selected according to a correlation analysis between the outcome variables and the additional information contained in the survey. Results confirm that right-leaning ideology is positively associated with self-interest (*β* = 0.033, *t* = 4.30, *P* <  0.001) and national generosity (*β* = 0.014, *t* = 2.13, *P* = 0.033) and negatively associated with international generosity (*β* = −0.047, *t* = −10.10, *P* <  0.001). All variables have been normalized between 0 and 1; thus, for example, very left-leaning participants are less self-interested than very right-leaning participants by about 3.3% points, a tiny effect in line with previous work on political differences in cooperation (9). We refer to the OSF repository for additional analyses where we control for the severity of the pandemic, compare representative vs convenience samples, and illustrate the stability of the results across countries.

**Table 1.** Amount kept for oneself (self-interest), donated to a national charity (national), and donated to an international charity (international)

	(1)	(2)	(3)	(4)	(5)	(6)	(7)	(8)	(9)
	Self-interest	National	International	Self-interest	National	International	Self-interest	National	International
Political ideology	0.083*** (0.007)	0.001 (0.006)	−0.086*** (0.005)	0.042*** (0.007)	0.018** (0.006)	−0.062*** (0.004)	0.033*** (0.008)	0.014* (0.006)	−0.047*** (0.005)
Constant	0.445*** (0.004)	0.338*** (0.003)	0.219*** (0.003)	0.465*** (0.004)	0.329*** (0.003)	0.207*** (0.002)	0.782*** (0.015)	0.008 (0.013)	0.210*** (0.010)
Controls	No	No	No	No	No	No	Yes	Yes	Yes
Country fixed effects	No	No	No	Yes	Yes	Yes	Yes	Yes	Yes
Observations	46,481	46,500	46,372	46,481	46,500	46,372	43,499	43,501	43,467
$R^{2}$	0.003	0.001	0.008	0.122	0.074	0.163	0.155	0.116	0.183

Notes: Columns (1) to (3) report OLS regressions with robust SEs in parentheses. Columns (4) to (6) take into account the hierarchical nature of the data by adding country-level fixed effects. Columns (7) to (9) control for gender, age, employed, student, collective narcissism, national identity, individual narcissism, moral circle, moral cooperation, open-mindedness, COVID-19 conspiracy beliefs, health condition, and self-ladder. All variables were normalized between 0 and 1. *P <  0.05, **P <  0.01, and ***P <  0.001.

**Fig. 1.**
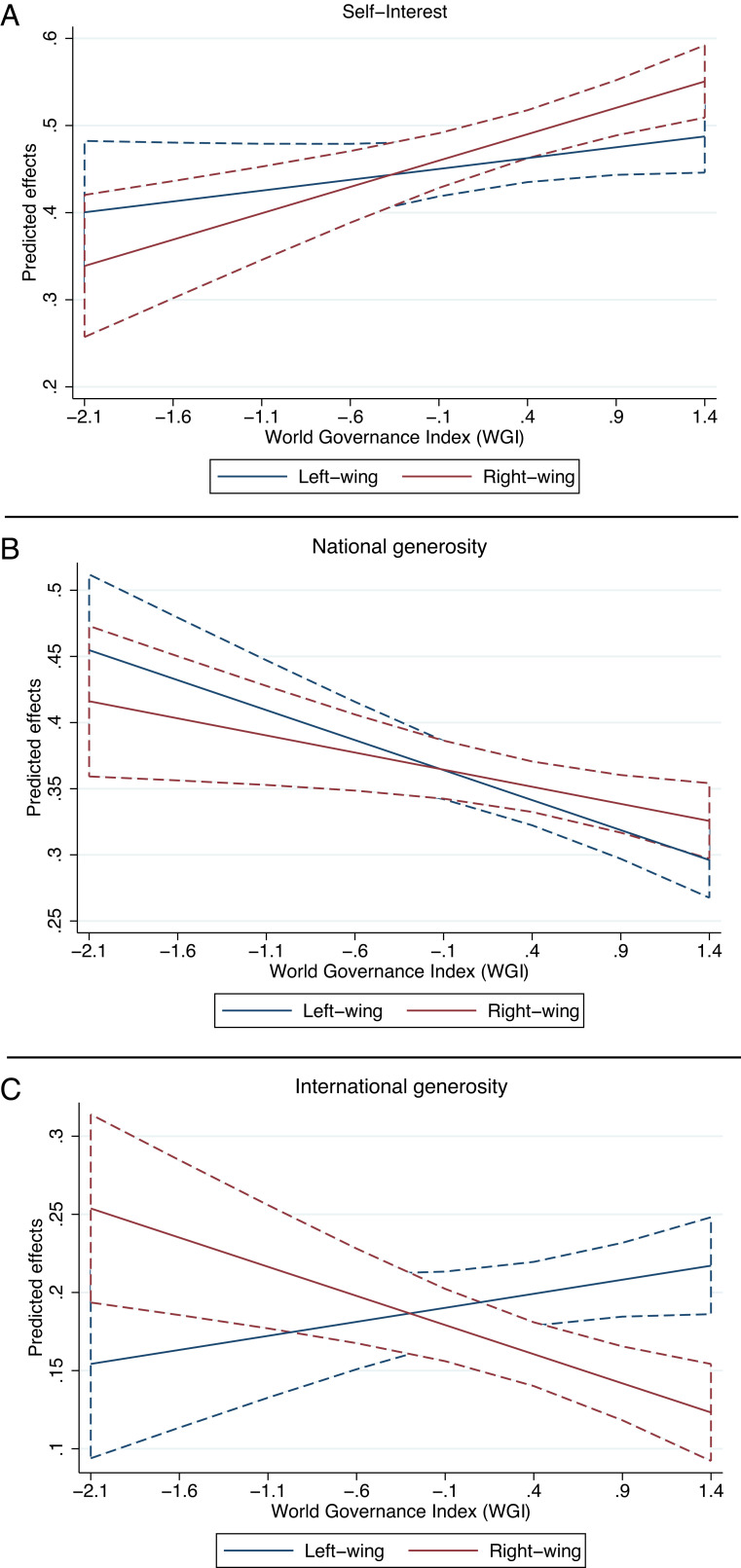
Linear predictions based on multilevel mixed-effects models of the Worldwide Governance Indicator (WGI) interacted with political ideology predicting (*A*) self-interest, (*B*) donations to a national charity, and (*C*) donations to an international charity. The blue lines correspond to political ideology = 0.1 on a normalized scale between 0 (very left-leaning) and 1 (very right-leaning); red lines to political ideology = 0.9. CIs are at the 95% level.

We also examine if the relationship between political ideology and generosity may be influenced by country-level factors. We run a moderation analysis, considering the quality of governance as a source of possible cross-country variation. We use the Worldwide Governance Indicator (WGI), which incorporates six different dimensions of governance: voice and accountability, political stability and absence of violence/terrorism, government effectiveness, regulatory quality, rule of law, and control of corruption. Fig. 1 shows the mixed-effects linear predictions derived from the interaction between WGI and political ideology for the three outcome variables. Thus, when the quality of governance increases, individuals increase self-interest, with the trend being steeper for right-leaning individuals (Panel *A*, *β* = 0.045, *z* = 5.44, *P* <  0.001), and decrease national generosity, with the trend being flatter for right-leaning individuals (Panel *B*, *β* = 0.024, *z* = 3.52, *P* <  0.001). Panel (*C*) illustrates how right- and left-leaning individuals adopt opposite behaviors toward an international charity when the quality of governance increases: Right-leaning individuals tend to donate less; left-leaning ones tend to donate more (*β* = −0.069, *z* = −13.75, *P* <  0.001). The results are robust to using each index as a separate measure of the quality of governance and to including all the controls.

## Discussion

Using a global dataset, we found that left-leaning people, compared to right-leaning people, tend to be more generous in general and toward an international charity. We also observe that right-leaning people tend to donate more at the national level. These results are robust to the inclusion of several controls.

We studied the heterogeneity of the effects across countries by making use of an indicator of the quality of governance. We showed that the quality of governance moderates the three correlations between political ideology and the various measures of generosity. These results suggest a double substitution effect. On the one hand, as the quality of governance increases, left-leaning people may tend to embrace either the universalist or the individualist values typically brought forward by countries with high quality of governance; these values put little emphasis on local boundaries (14). On the other hand, as the quality of governance increases, right-leaning people may react negatively to the universalist values, through a cultural backlash (2), and therefore increase only their individualism.

We contribute to previous research in multiple ways. Using a unilateral decision, as opposed to a strategic one (9), we can conclude that political ideology also affects preferences. This is in line with previous work finding a correlation between right-wing ideology and pro-self-orientation (15). Yet, we make a step forward measuring national and international forms of generosity at a global level. This allows us to study the moderation effect of quality of governance and to shed light on the underlying mechanisms driving the results.

In sum, this work provides insights about the link between political ideology and altruistic preferences around the globe and about how this relationship varies as a function of the quality of governance.

## Materials and Methods

The individual-level data used in this study have been collected by the International Collaboration on Social and Moral Psychology of COVID-19. The survey was approved by the Ethics Board at the University of Kent. All relevant ethical regulations were followed, and all participants were asked to give informed consent. The country-level data leading to the Worldwide Governance Indicator have been downloaded from the World Bank. Details can be found in *SI Appendix*.

## Supplementary Material

Appendix 01 (PDF)

## Data Availability

The dataset and the STATA source code for all the analyses are available on the Open Science Framework repository: https://doi.org/10.17605/osf.io/xtmwz (16). Previously published data were used for this work (13) OSF Repository: https://doi.org/10.17605/osf.io/tfsza (17).
