# Supplementary material for: Political ideology and generosity around the globe
Source: Proc Natl Acad Sci U S A. 2023 Apr 5;120(15):e2219676120. doi: 10.1073/pnas.2219676120 (PMC10104563; doi:10.1073/pnas.2219676120)
Supplement: Supplementary file 1 — Appendix 01 (PDF) [file pnas.2219676120.sapp.pdf]

PNAS
www.pnas.org

Supplementary Information for

Political Ideology and Generosity Around the Globe

Veronica Pizziol, Xhiselda Demaj, Roberto Di Paolo, and Valerio Capraro

Corresponding Author: Veronica Pizziol
E-mail: veronica.pizziol@imtlucca.it

This PDF file includes:

SI References

1. Detailed materials and methods

Materials. In this work, we employ the International Collaboration on the Social and Moral Psychology of COVID-19 (ICSMP COVID-19) dataset (1). The *Collaboration* aimed at gathering data worldwide on psychological factors that might be related to the COVID-19 pandemic response. It was launched in April 2020, and more than 200 scholars recruited samples in their own countries. Official data were collected between April 22nd and June 3rd, 2020. The survey was created in English and then disseminated to each national team, which was responsible for translating it into their local language (using the standard forward-backward method).

A total of 51,402 individuals from 77 samples across 69 countries participated in the survey. National teams collected 33 representative samples with respect to age and gender. The remaining data were drawn from convenience samples. The cleaned dataset is available on the Open Science Framework (doi.org/10.17605/osf.io/tfsza).

In the current project, we were interested in the correlation between political ideology and generosity. Therefore, we conducted a correlational analysis to see which variables were correlated to our outcome measures to be used as controls. We built a new dataset that can be found on the Open Science Framework repository (https://osf.io/xtmwz/?view_only=7b1e6329f104495b90e9f60fc5a6eabc) that contains the variables of interest, and that will be used for the analysis in the current work. The list of variables, along with their definition, is reported in the next section.

To complete our analysis, we also employed the Worldwide Governance Indicator (WGI). The WGI is a governance index for more than 200 countries that incorporates six relevant dimensions of governance through 1996-2021. It is constructed as the average between the following variables: Voice and Accountability, Political Stability and Absence of Violence/Terrorism, Government Effectiveness, Regulatory Quality, Rule of Law, and Control of Corruption (more information can be found at the following link: https://info.worldbank.org/governance/wgi/Home/Documents).

Methods. The variables used in this study are listed below. Regarding the ICSMP COVID-19 dataset, most of the variables were collected on an 11-point scale, where 0 refers to "strongly disagree", 10 is equivalent to "strongly agree", and 5 identifies "neither disagree nor agree". In some cases, when more appropriate, other response scales were used (e.g., a 0-100% response scale was applied in the generosity measure). To ensure participants anonymity, no data that would allow their identification were collected.

Generosity was measured as the proportion over 100 of the daily wage in the corresponding country that a participant would keep for themselves (*Self-interest*) versus how much they would give to a national charity (*National generosity*) or to an international charity (*International generosity*).[*]

Political ideology was measured using a single item, "Overall, how would you describe yourself in terms of political ideology?", on a scale ranging from 0, corresponding to "very left-leaning", to 10, corresponding to "very right-leaning" (5).

National identity was constructed by combining the two items: "I identify as [nationality]" and "Being a [nationality] is an important reflection of who I am" (6).

Collective narcissism was measured using three items of the original Collective Narcissism scale (7): "[My national group] deserves special treatment", "Not many people seem to fully understand the importance of [my national group]", "I will never be satisfied until [my national group] gets the recognition it deserves".

Moral circle was based on the moral circle scale (8). Participants indicated the extent of their moral circle across 16 different entities (human and not), which might deserve moral concern, ranging from the closest family to all existing things.

Moral cooperation was measured using the relevance sub-scale of the Morality-as-Cooperation Questionnaire (MAC-Q, (9)). It quantifies the extent to which each of the seven aspects of cooperation is relevant when making moral decisions. The final variable was the average of the seven items.

Open-mindedness was measured with a six-item scale from the Multidimensional measure of intellectual humility (10), which reflects the acknowledgment of one's knowledge limitations and openness to learning new things.

COVID-19 conspiracy beliefs was measured as the average of four 11-point scale items, i.e., "The coronavirus (COVID-19) is a bioweapon engineered by scientists"; "The coronavirus (COVID-19) is a conspiracy to take away citizens rights for good and establish an authoritarian government"; "The coronavirus (COVID-19) is a hoax invented by interest groups for financial gains"; "The coronavirus (COVID-19) was created as a cover-up for the impending global economic crash".

Self ladder was constructed from the responses to the MacArthur scale of subjective social status (11) as a measure of the subjective socioeconomic status of the participants. Participants placed themselves on an 11-rung ladder, with the bottom rung representing the individuals who are worst off and the top rung representing individuals who are best off.

Health condition was measured by asking participants to rate their physical health on the day of the study on an 11-point scale from "Extremely bad" to "Extremely good".

Gender was constructed by assigning a value equal to 1 to the participants identifying as "Female", and a value equal to 0 to those identifying as "Male".

Age was measured as the self-reported age of the participants at the time when the study was taken.

Employed was constructed by assigning a value equal to 1 to the participants who reported being employed at the time of the study and assigning 0 otherwise.

Student was constructed by assigning a value equal to 1 to the participants who reported being students at the time of the study and assigning 0 otherwise.

[*] The generosity measure did not mention any specific charity and was hypothetical; previous work found no difference between hypothetical and incentivized national parochialism (2–4).

COVID-19 cases was defined as the average number of confirmed COVID-19 cases per day per 100.000 residents in a specific country during data collection. Data were downloaded from (12).

COVID-19 deaths was defined as the average number of confirmed COVID-19 deaths per day per 100.000 residents in a specific country during data collection. Data were downloaded from (12).

Representative sample is a dummy variable that takes value equal to 1 for those samples that are representative with respect to sex and age and takes value 0 otherwise.

Voice and Accountability is an index capturing perceptions of the extent to which a country's citizens can participate in selecting their government, as well as freedom of expression, freedom of association, and free media.

Political Stability and Absence of Violence/Terrorism measures perceptions of the likelihood of political instability and/or politically motivated violence, including terrorism.

Government Effectiveness captures perceptions of the quality of public services, the quality of the civil service and the degree of its independence from political pressures, the quality of policy formulation and implementation, and the credibility of the government's commitment to such policies.

Regulatory Quality is an index capturing perceptions of the ability of the government to formulate and implement sound policies and regulations that permit and promote private sector development.

Rule of Law measures perceptions of the extent to which agents have confidence in the rules of society, particularly the quality of contract enforcement, property rights, the police, and the courts, as well as the likelihood of crime and violence.

Control of Corruption captures perceptions of the extent to which public power is exercised for private gain, including both small and big forms of corruption, as well as the "capture" of the state by elites and private interests.

2. Replication materials

The dataset and the STATA source code used for all the analyses are available via OSF repository:
https://osf.io/xtmwz/?view_only=7b1e6329f104495b90e9f60fc5a6eabc

References

1. F Azevedo, et al., Social and moral psychology of covid-19 across 69 countries. *Nat. Sci. Data* (forthcoming).
2. D Balliet, J Wu, CK De Dreu, Ingroup favoritism in cooperation: a meta-analysis. *Psychol. Bull.* **140**, 1556 (2014).
3. A Romano, M Sutter, JH Liu, T Yamagishi, D Balliet, National parochialism is ubiquitous across 42 nations around the world. *Nat. Commun.* **12**, 1–8 (2021).
4. A Romano, M Sutter, JH Liu, D Balliet, Political ideology, cooperation and national parochialism across 42 nations. *Philos. Transactions Royal Soc. B* **376**, 20200146 (2021).
5. JT Jost, CM Federico, JL Napier, Political ideology: Its structure, functions, and elective affinities. *Annu. Rev. Psychol.* **60**, 307–337 (2009).
6. T Postmes, SA Haslam, L Jans, A single-item measure of social identification: Reliability, validity, and utility. *Br. J. Soc. Psychol.* **52**, 597–617 (2013).
7. AG de Zavala, A Cichocka, R Eidelson, N Jayawickreme, Collective narcissism and its social consequences. *J. Pers. Soc. Psychol.* **97**, 1074 (2009).
8. A Waytz, R Iyer, L Young, J Haidt, J Graham, Ideological differences in the expanse of the moral circle. *Nat. Commun.* **10**, 1–12 (2019).
9. OS Curry, MJ Chesters, CJ Van Lissa, Mapping morality with a compass: Testing the theory of morality-as-cooperationwith a new questionnaire. *J. Res. Pers.* **78**, 106–124 (2019).
10. M Alfano, et al., Development and validation of a multi-dimensional measure of intellectual humility. *PloS One* **12**, e0182950 (2017).
11. NE Adler, ES Epel, G Castellazzo, JR Ickovics, Relationship of subjective and objective social status with psychological and physiological functioning: Preliminary data in healthy, white women. *Heal. Psychol.* **19**, 586 (2000).
12. E Mathieu, et al., Coronavirus pandemic (covid-19). *Our World Data* (2020) https://ourworldindata.org/coronavirus.
